# Sleep in Healthy and Pathological Aging

**DOI:** 10.3390/brainsci14020128

**Published:** 2024-01-26

**Authors:** Maurizio Gorgoni, Luigi De Gennaro

**Affiliations:** 1 Department of Psychology, Sapienza University of Rome, 00185 Rome, Italy; luigi.degennaro@uniroma1.it; 2 IRCCS Fondazione Santa Lucia, 00179 Rome, Italy

## 1. Introduction

Human sleep physiology is strongly affected by age. Indeed, healthy aging is characterized by relevant changes in sleep habits, sleep macrostructure, and electroencephalographic (EEG) sleep features [1]. Moreover, the diurnal modulation of sleepiness and vulnerability to sleep loss change with age [1], and the prevalence of several sleep disorders shows an age-related increase [2]. Sleep changes in older individuals may be influenced by several factors, such as an increase in different pathological conditions (and the parallel increase in medication use, with a high frequency of polypharmacotherapy) and age-related environmental and psychosocial changes [2]. However, the presence of sleep and sleep-related alterations in older individuals can have relevant functional consequences in terms of reduced physical and mental health and quality of life [3], as well as direct effects on cognitive functioning [1].

It is worth noting that several age-related pathological conditions are characterized by significant sleep alterations. In particular, sleep/wake cycle disruption, sleep pattern changes, and increased sleep disorders are typical in neurodegenerative disorders such as Alzheimer’s disease (AD) and Parkinson’s disease (PD) (e.g., [4,5]). Crucially, a growing body of evidence suggests that specific sleep alterations may predict neurodegenerative processes and may play a role in the etiopathogenesis of AD [5]. In this regard, the possible relevance of sleep-based interventions in the pre-clinical stage and during the course of AD pathology has been highlighted [6].

The growing literature underlines the need for a better understanding of the causes, consequences, and functional significance of age-related sleep changes; their relationship with pathological conditions; and their possible role in clinical settings, especially considering that the aging population is progressively growing. Starting from this premise, the present Special Issue aimed to collect articles focused on sleep in healthy and pathological aging using different methodologies. In this editorial, we highlight the main contents of each contribution to this Special Issue in order to encourage the reader to explore them in detail.

## 2. Special Issue Overview

The first paper of this Special Issue (contribution 1) provides evidence on the spatiotemporal EEG pattern of the sleep onset (SO) process in healthy older adults. Although a consolidated body of evidence points to progressive local frequency-specific changes during the wake/sleep transition in humans [7], with specific age-related features in children [8], a description of the regional EEG pattern during the SO process in older adults is lacking. This contribution describes for the first time the EEG topography of the SO process in older adults, and it appeared to be similar to that observed in younger adults but with some age-related peculiarities: (a) the global power increase in the delta/theta range did not encompass the 7 Hz frequency bin; (b) the power in the alpha range exhibited a complex pattern of frequency-specific post-SO modifications; (c) after the SO process, sigma activity showed only a slight increase, and the fastest bins in the sigma range exhibited a fronto-temporal power reduction. A direct comparison between younger and older adults showed a wide aging-related reduction in the relative delta power and delta/beta ratio during both pre- and post-SO periods, except for in the right occipitotemporal region, which showed the absence of group differences before SO and an increased delta power and delta/beta ratio in older adults after SO. These findings suggest a reduced homeostatic regulation and a greater cortical arousal during the wake/sleep transition in healthy older individuals, which may be a target of future intervention strategies to support the SO process in this population.

The second article (contribution 2) starts from evidence showing that sleep EEG features are associated with measures of cortical thickness in healthy young and older individuals [9,10] to assess the hypothesis that specific sleep EEG alterations observed in AD [5] may be associated with the structural brain changes that characterize these patients. Specifically, the study assesses the relationship between sleep EEG features collected during a polysomnographic (PSG) recording and cortical thickness measurements made with magnetic resonance imaging (MRI) in a group of 23 AD patients. The results show that all the EEG indices considered were associated with the thinning of the right precuneus. Moreover, fronto-central NREM sigma power exhibited an inverse correlation with the thinning of the left entorhinal cortex. The observed frontopolar and temporal increase in delta activity was related to frontal, temporal, and parietal atrophy and the mean thickness of the right hemisphere. Although the small sample size and the absence of a group of healthy older individuals limit the possibility of generalizing these results, the study suggests that specific sleep EEG indices may represent compensatory mechanisms in local sleep-dependent memory consolidation processes or markers of progressive cortical neurodegeneration in AD.

The third paper (contribution 3) keeps the focus on AD but examines the animal model. Specifically, the authors investigate the hypothesis that sleep disturbances and circadian rhythm alterations in AD may have consequences on memory processes, assessing the effect of training time schedules (early morning or midday) on 42 12-month-old male 3xTg-AD mice modeling advanced disease stages, using learning paradigms in the Morris water maze. The authors also considered the effect of chronic treatment with anti-AD compounds. The results show that performance in the Morris water maze was sensitive to the training time schedule. Spatial reference and visual perceptual learning and memory performance were weakened at midday, after 4 h of the non-active phase. However, early-morning-trained littermates, slowing down from their active phase, showed better performance, and they used goal-directed strategies and non-search navigation described for normal aging. Considering the effect of treatment, a novel multitarget anticholinesterasic compound (AVCRI104P3) had greater benefits on performance than the in vitro equipotent dose of AChEI huprine X, with both showing streamlined effects independently of the schedule and moderate effects on anxiety. These findings suggest the need to better understand the effect of time schedules on the response to task demands and the factors that modulate drug effectiveness.

The article by Figorilli and coworkers (contribution 4) is the first narrative review of this Special Issue, focused on the neurophysiological aspects of REM sleep behavior disorder (RBD), which, in isolation, is considered an early manifestation of neurodegenerative processes, particularly synucleinopathies [11]. In this extensive narrative review, the authors first provide a detailed description of the neural network underlying REM sleep and, specifically, REM atonia, and then they focus on REM sleep without the atonia (RSWA) phenomenon that characterizes RBD. The evidence provided by the authors suggests that RBD alters the overall neurochemical balance of the central nervous system, highlighting the role of the complex interactions between the brainstem and other brain structures, as well as the possibility of detecting them through neurophysiological markers. They describe evidence from PSG studies and consider the current methods for RSWA detection and quantification. Then, the authors provide a description of the recent findings obtained in RBD using wake and sleep EEG, transcranial magnetic stimulation, and vestibular evoked myogenic potentials, suggesting that their integration with clinical, neuroimaging, and sleep-related data may help to understand the neurophysiological alterations of RBD and open new pathways for future treatment strategies. The authors also point to the relevance of the neurophysiological assessment in RBD; its role in the detection of neurodegeneration; and its relationship with other biomarkers for the diagnosis, prognosis, and monitoring of this condition. Finally, the authors propose some relevant areas for future research in this field.

The second narrative review of this Special Issue (contribution 5) provides an in-depth description of the mechanisms and processes of sleep/wake regulation in normal aging. First, the authors consider the main age-related changes in sleep organization and structure, describing the effects of age on sleep timing, duration, initiation, maintenance, and macro- and micro-structures. Then, they describe how the interaction between the circadian and homeostatic processes regulates the sleep/wake cycle, highlighting how these processes are affected by aging. Finally, they review evidence on sleepiness and vulnerability to sleep deprivation in healthy aging.

Starting from evidence showing that sleep disorders can be considered risk factors for dementia [12,13,14], the article by Legault and coworkers (contribution 6) reviews individual vulnerability and protective factors that may play a role in the relationship between obstructive sleep apnea (OSA) and cognitive decline, with a particular focus on mild cognitive impairment (MCI) and AD. The authors define the potential processes that link OSA and cognitive decline, as well as providing the main epidemiological evidence. Then, they focus on the individual characteristics that can moderate this association: age, sex, menopause, obesity, diabetes mellitus, hypertension, cardiovascular diseases, smoking, excessive alcohol consumption, depression, air pollution, the Apolipoprotein E ε4 (ApoE4) allele, physical activity, and cognitive reserve. In addition to underlining the clinical relevance of the identification of these protective and vulnerability factors, the authors propose several future directions in this research field.

The seventh paper (contribution 7) presents a systematic review and meta-analysis aimed at investigating the role of physical therapy exercises in a clinical setting on sleep disorders in patients with neurological disorders. Indeed, sleep may be altered under conditions of hospitalization [15,16], and sleep disorders may negatively affect the outcome of neurorehabilitation [17]. However, physical exercise has a beneficial effect on sleep disorders [18]. Therefore, it could be useful to identify specific protocols to support conventional neurorehabilitation programs. The authors identified 10 articles on this topic, and 6 of them were included in the meta-analysis. The main results suggest that physical therapy exercises may have a beneficial effect on sleep disorders in neurological patients, but the generalization of these findings is limited by the relatively small number of studies and the heterogeneity of the proposed interventions. The authors point to the need for a higher methodological quality of future studies in this field.

The last article (contribution 8) is a systematic review on the age-related effects of sleepiness on driving performance. Sleepiness can account for 10–20% of road traffic injuries [19,20]. While the absolute number of crashes is low in older individuals, the percentage of older individuals with a driving license is growing, and the frequency of crashes per mile driven increases at around 65 years of age [21]. However, the role of sleepiness in driving performance in older individuals has been rarely investigated. Therefore, the systematic review aims to discuss studies on the relationship between sleepiness, aging, and driving skills. Ten articles were identified and selected for the systematic assessment. The main findings suggest that older drivers are less vulnerable to sleep loss and sleepiness-related driving impairments than young drivers, probably due to age-related differences in lifestyle. Moreover, it has been proposed that older drivers may alter their driving to avoid dangerous situations. However, several studies suggest that older adults may underestimate their sleepiness level, and the time of day of the assessment may also play a role. Finally, the reviewed literature is scarce and characterized by several limitations, and future perspectives are highlighted in the article.

## 3. Conclusions

Overall, the contributions to this Special Issue consider the relationship between sleep and aging from different perspectives and with the use of distinct methodologies. The sleep changes in healthy aging are considered in three articles, increasing our knowledge on age-related local sleep peculiarities by focusing on the SO process (contribution 1); describing changes in sleep regulation processes with age (contribution 5); and highlighting the effect that sleep-related changes may have on daily life, with a focus on the relationship between sleepiness and driving performance (contribution 8).

Significant attention has been paid to AD, from the description of the relationship between local sleep EEG and cortical thickness in these patients (contribution 2) to the assessment of the effect of training time schedules on learning performance in the AD animal model (contribution 3) and the review of the evidence on the individual vulnerability and protective factors that mediate the relationship between OSA and AD (contribution 6). Taken together, these articles underline the strong relevance of sleep research in the field of AD and highlight the need for new knowledge on the relationship between sleep alterations and dementia.

A specific focus on the neurophysiological aspects of RBD is provided by contribution 4. Increasing our understanding of RBD neurophysiology is crucial for providing new possible biomarkers to improve the diagnosis and the possibility of monitoring and predicting the evolution of the disorder, especially considering its relationship with neurodegenerative disorders.

Finally, one article considered the role of sleep in a clinical setting, reviewing the evidence on the effect of physical therapy exercises on sleep disorders in patients with neurological disorders (contribution 7). Together with other recent findings, the observed results encourage the consideration of sleep in the aging population as a possible target of intervention.
